# Encapsulation of docetaxel in oily core polyester nanocapsules intended for breast cancer therapy

**DOI:** 10.1186/1556-276X-6-630

**Published:** 2011-12-14

**Authors:** Ibrahima Youm, Xiao Yan Yang, James B Murowchick, Bi-Botti C Youan

**Affiliations:** 1 Laboratory of Future Nanomedicines and Theoretical Chronopharmaceutics, Division of Pharmaceutical Sciences, University of Missouri-Kansas City, 2464 Charlotte Street, Kansas City, MO, 64108, USA; 2 Department of Geosciences, University of Missouri-Kansas City, 420 Flarsheim Hall, 5110 Rockhill Rd., Kansas City, MO, 64110, USA

**Keywords:** docetaxel, polylactide, emulsion diffusion, nanocapsules, drug loading

## Abstract

This study is designed to test the hypothesis that docetaxel [Doc] containing oily core nanocapsules [NCs] could be successfully prepared with a high percentage encapsulation efficiency [EE%] and high drug loading. The oily core NCs were generated according to the emulsion solvent diffusion method using neutral Labrafac CC and poly(*d, l*-lactide) [PLA] as oily core and shell, respectively. The engineered NCs were characterized for particle mean diameter, zeta potential, EE%, drug release kinetics, morphology, crystallinity, and cytotoxicity on the SUM 225 breast cancer cell line by dynamic light scattering, high performance liquid chromatography, electron microscopies, powder X-ray diffraction, and lactate dehydrogenase bioassay. Typically, the formation of Doc-loaded, oily core, polyester-based NCs was evidenced by spherical nanometric particles (115 to 582 nm) with a low polydispersity index (< 0.05), high EE% (65% to 93%), high drug loading (up to 68.3%), and a smooth surface. Powder X-ray diffraction analysis revealed that Doc was not present in a crystalline state because it was dissolved within the NCs' oily core and the PLA shell. The drug/polymer interaction has been indeed thermodynamically explained using the Flory-Huggins interaction parameters. Doc release kinetic data over 144 h fitted very well with the Higuchi model (*R*^2 ^> 0.93), indicating that drug release occurred mainly by controlled diffusion. At the highest drug concentration (5 μM), the Doc-loaded oily core NCs (as a reservoir nanosystem) enhanced the native drug cytotoxicity. These data suggest that the oily core NCs are promising templates for controlled delivery of poorly water soluble chemotherapeutic agents, such as Doc.

## Background

In cancer therapy, most of the proposed formulations present certain drawbacks related to the formulation properties including low drug loading, toxicity, and unsuitable release pattern. An ideal formulation should provide biocompatible nanosized particles and high drug loading with sustained-release characteristics. This allows releasing the drug in the target site in its therapeutic concentration and preventing drug inefficiency and side effects. The current research was aimed to prepare highly loaded docetaxel [Doc] oily core nanocapsules [NCs].

Doc, a semisynthetic analog of paclitaxel, is an extract from the needles of the European yew tree *Taxus baccata *[1]. It is prepared by chemical modification of 10-deacetylbacattin III, an inactive precursor compound, and then isolated [2]. Doc is a highly potent, cytotoxic, and antimitotic agent used in the treatment of various types of cancers, including metastatic breast, ovarian, prostate, advanced non-small-cell lung, head/neck, and advanced gastric cancers by inhibiting the microtubule depolymerization of free tubulin [3,4]. Due to its poor water solubility (10 to 20 μg/l), polysorbate 80 has been markedly used to improve the aqueous solubility of Doc [5-7]. This currently available, marketed formulation has been associated with the absence of selectivity for target tissues, serious dose limiting toxicities, and hypersensitivity reactions, as well as sensory and motor neuropathies that are sometimes severe and irreversible. Previously, various alternative formulations, including NCs, pegylated liposomes, targeted immunoliposomes, Doc-fibrinogen-coated olive oil droplets, and cyclodextrins [8-15] have been intensively developed for the delivery of Doc. However, the nanosized polymeric nanoparticles represent promising drug-delivery systems which have some advantages such as biodegradability, good biocompatibility, non-toxicity, higher stability, and controlled drug delivery. Polymeric nanoparticles (nanospheres and NCs) not only maintain a prolonged circulation time in the body (especially when pegylated) by avoiding the reticuloendothelial system, but also can extravagate and accumulate into the tumor tissue. This is likely due to the reliance of these nanoparticles on passive accumulation through enhanced permeability and retention, which is highly dependent on adequate blood flow to the tumor [16].

According to the literature, the NCs correspond to nanostructures with polymeric wall enveloping an oily core, whereas the nanospheres consist of a polymeric matrix [17]. There is increasing scientific evidence supporting the notion that certain lipids are able to inhibit both presystemic drug metabolism and P-glycoprotein-mediated drug efflux [18]. Several polymers have been proposed as nanocarriers for drug delivery systems. For example, poly(*d, l*-lactic acid) and poly(ε-caprolactone) [PCL] have been extensively used as nanocarriers because of their excellent biocompatibility and biodegradability. These polyesters have been approved by the US Food and Drug Administration and are the most widely used commercial polymers for drug delivery [19,20]. Presently, the only available Doc formulations for clinical use consists of intravenous [IV] solutions containing Tween 80^® ^(Sanofi-aventis, Bridgewater, NJ, USA). These solutions namely Taxotere^® ^and Docetaxel^®^, 10 to 20 mg/ml, are administered IV at a dose ranging from 60 to 100 mg/m^2 ^over 1 h every 3 weeks [21]). However, such high doses and a long term medication schedule may produce more severe side effects [22]. One of the reasons could be ascribed to the composition of the formulation and the poor control of the drug release rate. Therefore, the development of compatible polymer-based nanocarriers with high drug loading might be a helpful subject in cancer research.

The current research was aimed to prepare highly loaded Doc oily core NCs. However, the relationship between drug and formulation excipients has also been investigated to better control the encapsulation process.

## Materials and methods

### Materials

Poly(*d, l*-lactide) [PLA], Resomer^® ^R206, Mw 125 kDa, with an inherent viscosity of 1.0 dl/g; PLA, Resomer^® ^R207, Mw 209 kDa, with an inherent viscosity of 1.5 dl/g; and PLA, Resomer^® ^R208, Mw 250 kDa, with an inherent viscosity of 1.8 dl/g were purchased from Boehringer Inc. (Ridgefield, CT, USA). PCL (Mw = 72 kDa) was kindly provided by Union Carbide (Danbury, CT, USA). Polyvinyl alcohol [PVA] (9 to 10 kDa and 30 to 70 kDa) and ethyl acetate were obtained from Sigma-Aldrich (St. Louis, MO, USA). Docetaxel or Doc was purchased from LC Laboratories (Woburn, MA, USA). Labrafac CC (caprylic/capric triglyceride, *d *= 0.945 g/cm^3^) was kindly supplied by Gattefosse Corporation (St-Priest, France) as a gift. For the high-performance liquid chromatography [HPLC] analysis, acetonitrile and methanol were supplied from Fisher (Thermo Fisher Scientific, Fair Lawn, NJ, USA). All the other reagents were of analytical grade and used without further purification.

### Preparation of the nanocapsules

#### Experimental design and general procedure for Doc-loaded nanocapsules

Fifteen formulations of Doc-loaded NCs were prepared by emulsion-diffusion method as previously described [19]. Briefly, the polyester (40 to 360 mg) was solubilized in water-saturated ethyl acetate (10 ml); then, the neutral oil (0.1 to 0.9 ml) containing Doc (2 to 18 mg) was further added to the organic mixture. The resulting solution was emulsified in 40 ml of PVA 2.5% to 5% (*w/v*) aqueous phase solution by homogenization (homogenizer, IKA ULTRA-TURRAX T-25, IKA Labortechnik, Staufen, Germany) at 8,000 rpm for 10 min. A large volume (200 ml) of deionized water was added dropwise into the previous solution to promote the diffusion of ethyl acetate into the aqueous phase. To remove the organic solvent, the nanosuspension was stirred under vacuum at 40°C Rotavapor^® ^RII (BUCHI Labortechnik AG, Flawil, Switzerland) for 30 min. NCs were recovered by ultracentrifugation at 12,000 rpm at 5°C for 30 min, washed twice with deionized water to remove the excess of PVA, and freeze-dried for 12 h (Labconco Corp. Kansas City, MO, USA). Blank NCs were prepared without Doc using the above method. The composition of the 15 formulations is listed in the first four columns of Table S1 in Additional file 1.

#### Screening for polyester selection based on the blank particle mean diameter

For the first screening test, a set of four biodegradable polymers, including PLA R206, PLA R207, PLA R208, and PCL, was used to obtain a suitable formulation based on the mean diameter of the obtained blank NCs. An amount of 200 mg of polyesters was solubilized in an organic phase containing 10 ml of water-saturated ethyl acetate and 0.5 ml of neutral oil. Each experiment was performed in triplicate.

#### Screening for stabilizer selection based on the blank particle mean diameter

Since PVA could be used as an emulsion stabilizer in the NCs' formulation process, it was important to first investigate the effect of PVA molecular weight (9 to 10 kDa and 30 to 70 kDa) on the blank NCs' mean diameter. Secondly, the influence of the PVA concentration (2.5% to 5%) on the particle mean diameter and polydispersity index [PDI] was studied.

### Physicochemical characterization of docetaxel-loaded nanocapsules

#### Particles' mean diameter and zeta potential analysis

The particle mean diameter and PDI of the Doc-loaded NCs were measured by dynamic light scattering [DLS] (Zetasizer Nano ZS series from Malvern Instruments Ltd., Worcestershire, UK) as recently reported [23]. The sample to be measured was appropriately diluted with water and briefly sonicated for 2 min. The measurement of each sample was completed at a scattering angle of 175°. Each measurement was done in triplicate, and the average effective diameter and polydispersity were recorded.

#### Drug loading and percent encapsulation efficiency of docetaxel-loaded nanocapsules

The freeze-dried NCs (2 mg) were dissolved in 0.1 ml of dichloromethane and diluted in methanol at the ratio of 1:14 *v/v*. After suitable dilutions, the amount of the encapsulated Doc was determined by HPLC (Waters Corporation, Milford, MA, USA) equipped with an UV detector at 230 nm. Isocratic flow of the mobile phase, composed of methanol/water/acetonitrile (30:30:40 *v/v/v*), was employed at a flow rate of 1.0 ml·min^-1 ^with a 10-μl injection volume. Doc separation was completed using an XBridge™ column C-18, at 4.6 × 150 mm and 3.5 μm (Waters Corporation, Milford, Massachusetts). The experimental Doc loading was quantified using the peak area of each NC formulation. Drug loading [DL] and percent encapsulation efficiency [EE%] were calculated according to Equations 1 and 2, respectively:

(1) $$\text{DL}=\frac{\text{Drug}\;\;\text{amount}}{\;\text{Polymer}\;\text{amount}+\text{drug}\;\;\text{amount}}\times \;100\%$$

(2) $$\text{EE\%}=\frac{\text{Experimental}\;\text{drug}\;\text{loading}}{\text{Theoretical}\;\text{drug}\;\text{loading}}\times \;100\%$$

#### Scanning electron microscopy analysis

Scanning electron microscopy [SEM] was used to assess the NCs' morphology. A droplet of NCs' suspension was put into a grid. The excess of the fluid was removed by wicking it off with an adsorbent paper, and then, it was visualized under a Hitachi S4700 cold-cathode field emission SEM [FESEM] (Hitachi High-Technologies Corporation, Minato-ku, Tokyo, Japan). The particles were sprinkled onto a stub covered with an adhesive conductive carbon tab, then sputter-coated with a fine layer of platinum metal. Then, the particles were imaged in the FESEM at 2 to 5 kV.

#### Transmission electron microscopy analysis

The selected samples were examined with a JEOL 1400 transmission electron microscope [TEM] (JEOL Ltd., Tokyo, Japan) and photographed digitally on a Gatan axis-mount 2 k × 2 k digital camera (Gatan, Inc., Pleasanton, CA, USA). The freeze-dried samples were put into a small mold, referred to as a BEEM capsule, and then imbedded in liquid epoxy resin (Epon-Araldite, Sigma-Aldrich Corporation, St. Louis, MO, USA). The resin was polymerized at 60°C for 2 days, and then, ultrathin 80-nm sections were cut on a Leica UCT ultramicrotome (Leica Microsystems Ltd., Milton Keynes, UK) with a Diatome diamond knife (Diatome, Hatfield, PA, USA). The sections were collected on 200-mesh copper grids and put into the TEM for imaging on a Gatan digital camera.

#### Powder X-ray diffraction pattern analysis

Powder X-ray diffraction [PXRD] analysis of the freeze-dried NCs was performed using a MiniFlex automated X-ray diffractometer (Rigaku, The Woodlands, TX, USA) at room temperature. Ni-filtered Cu Kα radiation was used at 30 kV and 15 mA. The diffraction angle covered from 2*θ *= 5° to 2*θ *= 60°, with a step size of 0.05°/step and a count time of 3 s/step (effectively 1°/min). The diffraction patterns were processed using Jade 8+ software (Materials Data, Inc., Livermore, CA, USA).

#### Refractive index measurement

The oil, water-saturated ethyl acetate, and ethyl acetate-saturated water refractive index values were measured experimentally at 25°C (Auto Abbe 10500 Refractometer; Reichert Analytical Instruments, Depew, NY, USA) using Milli-Q water (Millipore Co., Billerica, MA, USA) as a reference. A droplet of liquid was deposited on the prism surface. The obtained values are the average of five measurements.

#### In vitro drug release kinetics

This experiment was performed using the equilibrium dialysis method for 144 h. Specifically, a known amount of Doc-loaded oily core NCs (1 mg) was suspended in a dialysis bag (Spectra/Float-A-Lyzer, MWCO 3.5-5 kDa, Spectrum Laboratories Inc. Rancho Dominguez, CA, USA) containing 5 ml of phosphate buffer saline [PBS] (Sigma-Aldrich, St. Louis, MO, USA). The bag containing the NCs' suspension was placed in 40 ml of PBS. The system was placed in a shaking water bath (BS-06, Lab. Companion, Des Plaines, IL, USA) at 37°C with an agitation speed of 50 rpm. At predetermined time intervals, 500 μl of PBS solution was withdrawn from the immersion medium and replaced by the same volume of fresh medium. The cumulative percentage of drug released for each time point was calculated as a percentage of the total drug loading of the NCs tested. The quantitative analysis of the data obtained from the study was confirmed using Higuchi's kinetic model [24] to elucidate the mechanism of the drug release.

#### Solubility parameter determination

To predict the compatibility between the Doc (solubilizate) and polymer (solvent), the Flory-Huggins solubility parameter was evaluated [*χ*_sp_]. Our goal was to determine the interaction parameter *χ*_sp _[25] using the thermodynamic approach based on the extended Hildebrand solubility. According to Hildebrand, the solubility parameter [*δ*], defined as the square root of the cohesive energy density [CED], is equal to the energy of vaporization Δ*E*_v _per unit of molar volume (Equation 3) [26]. The solubility parameter is used to calculate *χ*_sp _using Equation 3:

(3) $$\delta ={\left(\text{CED}\right)}^{1∕2}={\left(\Delta E_{\text{v}}∕V_{\text{m}}\right)}^{1∕2}$$

Hansen modified the Hildebrand approach and divided *δ *into three components that take into account the force of the dispersion [*δ*_d_], the polarity [*δ*_p_], and the hydrogen bonds [*δ*_h_]. Therefore, *δ *is calculated using Equation 4:

(4) $$\delta_{t}^{2}=\delta_{d}^{2}+\delta_{p}^{2}+\delta_{h}^{2}$$

Equation 5 below was used to estimate the total Hildebrand solubility parameter based on the refractive index [*n*_D_], where *δ*_t _was the Hildebrand solubility parameter in (cal/cm^3^)^1/2^, and 304.5 was an empirically determined constant [27]. The refractometer was calibrated using pure water according to the instrument manual. Then, the total solubility parameter was calculated by using Equation 5:

(5) $$\delta_{\text{t}}=\sqrt{304.5\;\;\left(\frac{n_{\text{D}}^{2}-1}{n_{\text{D}}^{2}+2}\right)}$$

An interaction parameter with a value smaller than 0.5 (i.e., *χ*_sp _< 0.5) indicates that the solvent polymers are compatible. The interaction parameter can be calculated from the solubility parameters [28]. Considering the corresponding *δ*_t _for each component, the value of the interaction parameter (*χ*_sp_) can be estimated from the Hildebrand solubility parameters *δ*_s _and *δ*_p _(for solubilisant and polymer, respectively). On the basis of regular solution theory, the relationship between the Flory-Huggins interaction parameter and the solubility parameter is defined by using Equation 6:

(6) $$\chi_{\text{sp}}={\left(\delta_{\text{s}}-\delta_{\text{p}}\right)}^{2}.\;V_{\text{m}}∕R\;.\;T$$

where *V*_m _is the molar volume of the drug, *R *is the ideal gas constant (8.314 J·K^-1^·mol^-1^), and *T *is the temperature in Kelvin (293.15 K) [29].

#### In vitro evaluation of Doc-loaded nanocapsules' cytotoxicity

The NCs' cytotoxicity was evaluated using the SUM 225 cell line (Asterand, Inc., Detroit, MI, USA). The cytotoxicity after treatment of these cells with native Doc and Doc-loaded NCs at different equivalent drug concentrations (1 nM, 2.5 μM, and 5 μM) was evaluated by the lactate dehydrogenase [LDH] assay. The cells were plated at a density of 5 × 10^3 ^cells per well for 24 h in 96-well plates in a standard growth medium prior to exposure to the above materials. Blank NCs, and medium containing 0.5% dimethyl sulfoxide [DMSO] are used as negative controls, while Triton-X 1% (Sigma-Aldrich Corporation, St. Louis, MO) was used as a positive control and incubated for 24 h at 37°C in 5% CO_2_. After the treatment, the LDH reagents were used according to the manufacturer's instruction (Promega Life Sciences, Madison, WI, USA). The experimental results were expressed as mean values of six measurements (*n *= 6), and the cytotoxicity was calculated by the following formula:

(7) $$\text{Cytotoxicity}\;\left(\%\right)=\frac{\text{Experimental}-\text{Background}}{\text{Positive}\;\;-\text{Background}}\;\times \;\;100$$

where, experimental, background, and positive represent the fluorescence intensity of NC-treated wells, background wells (wells without cells), and positive control wells (cells treated with 1% of Triton X-100), respectively. The fluorescence intensity was detected by using a microplate reader (DTX 800 multimode microplate reader, Beckman Coulter, Brea, CA, USA) at an excitation wavelength of 560 nm and emission wavelength of 590 nm.

## Results and discussion

### Polymer selection based on the mean diameter of blank nanocapsules

**Figure 1:**
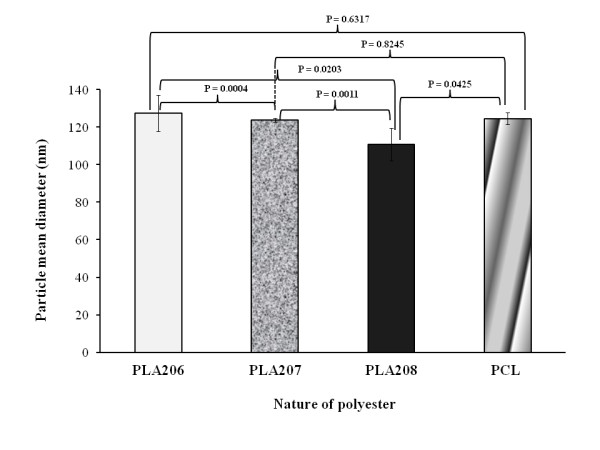
**Influence of the nature of biodegradable polyester on blank oily core nanocapsules mean diameter**. *n *= 3; S.D.: standard deviation between the three assays: PLA206 (105 kDa), PLA207 (209 kDa), PLA208 (250 kDa), and PCL (72 kDa).

This experiment was performed to find out the suitable polymer using small-sized NCs. Four polymers were initially screened including PLA R206, PLA R207, PLA R208, and PCL. Figure 1 shows the effect of different biodegradable polymers on the average diameter of blank NCs. The NCs' mean diameters were 127.5 ± 19.2 nm for NC-PLA206, 123.8 ± 0.9 nm for NC-PLA207, 110.8 ± 8.6 nm for NC-PLA208, and 124.6 ± 3.1 nm for NC-PCL. These findings indicated a statistically significant decrease of PLA NCs' diameters with increasing polymer molecular weights (*P *< 0.003, *T *test). Based on their smaller mean diameter, the NCs prepared with PLA R208 were selected for the subsequent studies. The results did not show any difference regarding the zeta potential values (*ζ *= -36.5 ± 9 mV) among the batches of NCs (data not shown). This high potential value also contributed to the stabilization of the nanosuspension.

### Stabilizer concentration and molecular weight effects on the blank particle mean diameter

**Figure 2:**
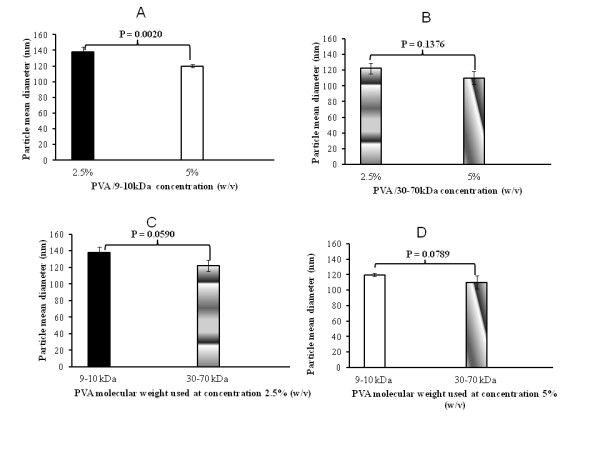
**Influence of PVA concentrations (A, B) and molecular weights (C, D) on the blank NC mean diameter**. This is detected by DLS (*n *= 3; S.D.: standard deviation between the three assays).

This preliminary experiment was performed to select the accurate PVA molecular weight and concentration with the goal of minimizing the particle size. Figure 2A, B, C, D shows that the NCs' mean diameter decreased with increasing both the PVA's molecular weight (9 to 10 kDa, to 30 to 70 kDa) and concentration (2.5% to 5%, *w/w*). Oppositely, previous studies have pointed out the increase of the PLGA NP mean diameter when the concentration of PVA was increased from 2% to 6% [30]. This effect was attributed to the increase of the external phase viscosity, which decreases the molecular diffusion rate and Ostwald ripening phenomenon. This revealed that the PVA concentration is not the only parameter governing the particle mean diameter. According to the overall results, PLA 208 and PVA (30 to 70 kDa, 5%) were finally selected for the following NC preparation.

### Preparation and characterization of docetaxel-loaded nanocapsules

For optimization purposes, 15 batches of PLA R208 Doc-loaded oily core NCs were prepared using the above method. As shown in Table S1 in Additional file 1, the lowest value of the NCs' diameter was obtained with F_6 _(115.6 nm), while the largest particle diameter was obtained with F_9 _(582.8 nm). The PDI ranged from 0.004 to 0.318 (F_14 _and F_9_, respectively). It was found that the particle mean diameter is strongly dependent to the polymer amount. Indeed, at a low PLA amount (40 mg), the particle mean diameter increases with increasing drug amount (see F_9 _versus F_10_, *P *< 0.0001). This might be explained by the fact that the lipophilic feature tends to decrease the leakage of the drug into the external aqueous medium, leading to improved drug content in the nanoparticles (which is consistent with the previous report) [19]. However, at a high PLA amount (360 mg), the particle mean diameter decreases with increasing drug amount (see F_4 _versus F_5_, *P *< 0.0001). The latter was not consistent with the commonly published report and might be a result of drug solubilization in the polymer matrix, leading to decrease the particle mean diameter. Thus, the solubilization capacity of PLA has a great importance in the preparation of the Doc-loaded NCs.

From Table S1 in Additional file 1, the data suggest that the NCs' mean diameter decreases with increasing oil content from 189 nm to 133 nm (see F_3 _versus F_13_, *P *< 0.0001). The results also show that at a low oil content, the drug level did not have any effect on the NCs' mean diameter, which is consistent with the previous study [31].

From Table S1 in Additional file 1, it appears that for most of the formulations, the PDI value was less than 0.05, indicating a monodispersity according to the National Institute of Standard [32]. However, the polydispersity seems to be increased with decreasing PLA amount (see F_13 _versus F_1_, from 0.005 to 0.296, *P *= 0.0078). This suggests that the PLA amount may contribute to ensure the NCs' monodispersity.

Table S1 in Additional file 1 lists also the EE% of the Doc-loaded NCs. Interestingly enough, the results showed that the EE% was mainly governed by the PLA content. A high PLA content led to a high EE% (see pairwise comparison: F_1 _to F_13_, F_3 _to F_9_, F_4 _to F_11_, F_5 _to F_10_, respectively). At a low PLA content, the EE% was decreasing regardless of the oil content (comparing F_9 _to F_10_, *P *= 0.0006). At a high oil content, a medium content of PLA is at least required to obtain a high EE% (see F_7 _and F_13_, *P *= 0.9038). The lowest EE% (F_9_, 65.3%) was obtained when the PLA and oil contents were both at the lowest level (40 mg and 0.1 ml, respectively). This is because the encapsulation process of hydrophobic drugs into these particles results from the interaction between the drug, polymers, and oil. Thus, the drug loading and EE% were found to depend on its solubility in the polymeric material, which is strongly related to the polymer composition, its molecular weight, the drug and polymer interaction, and the presence of end-functional ester or carboxyl groups [31]. These findings were consistent with a previous report [19]. Once the NCs' physicochemical properties have been analyzed, their size and morphology can be most directly monitored by various forms of electron microscopy.

### Physicochemical characterization of docetaxel-loaded nanocapsules

#### Morphological analysis

**Figure 3:**
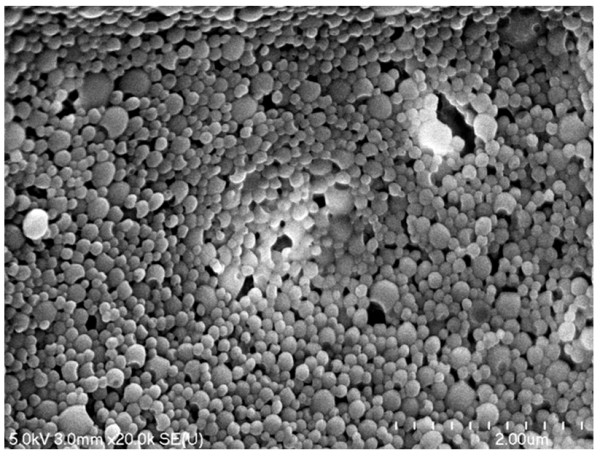
**Scanning electron micrographs of Doc-loaded oily core nanocapsules after freeze drying (F6)**. Scale bar represents 0.2 μm.

**Figure 4:**
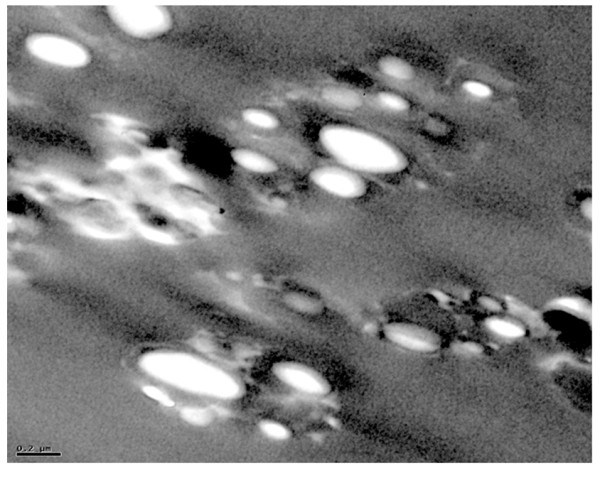
**Transmission electron micrographs of PLA 208 oily core nanocapsules after freeze drying (F6)**. Scale bar represents 0.2 μm.

The particle mean diameter and morphology were analyzed by SEM and TEM. Figure 3 shows a typical SEM picture of spherical NCs with smooth surfaces and undetectable free drug crystals. The NCs' size as estimated by SEM correlated well with the size measured by the DLS showing particles in a nanometric size range. The TEM analysis shows clearly a white and shiny oily core where Doc was well dissolved [33] (Figure 4). Therefore, it is necessary to investigate the drug structure inside the NCs.

#### Powder X-ray diffraction analysis

**Figure 5:**
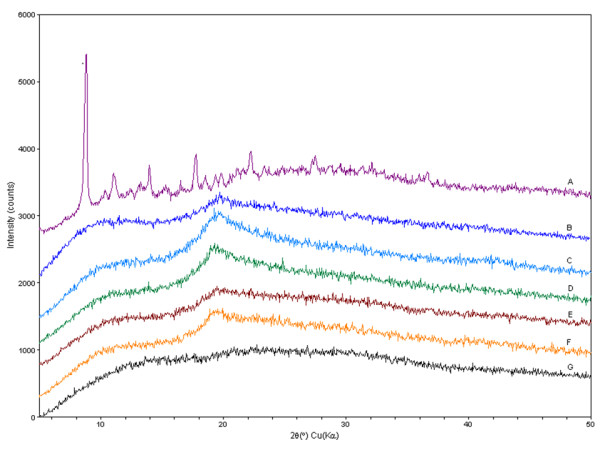
**PXRD pattern**. A, native Doc; B, F_13 _(PLA, 360 mg; Doc, 10 mg; oil, 0.9 ml); C, F_12 _(200 mg, 10 mg, 0.5 ml); D, F_6 _(200 mg, 18 mg, 0.9 ml); E, blank nanocapsules; F, native PVA; and G, native PLA 208.

This experiment aimed to characterize the crystallinity of Doc in the formulated NCs. Figure 5 shows the PXRD results of the formulations containing different amounts of Doc (F_6_, F_12_, and F_13_) and the individual native chemical compounds used in the tested formulations. The native Doc exhibited sharp and characteristic diffraction peaks at 8.72°, 10.28°, and 11.7°, which is consistent with a previous report [34]. The Doc crystals are orthorhombic. The unit cell parameters (at room temperature) are *a *= 39.9345 Å, *b *= 12.7749 Å, *c *= 8.6644 Å, with *V *= 4420.2 Å. The number of motifs (*Z*) per cell is 4, and the density of the crystal is 1.295 [35]. The native PLA is characterized by a broad diffraction peak, which was centered at 25°. The PXRD pattern of the native PVA shows a broad lump between 17° and 20° both in the native component and in the tested formulations, which suggests that the PVA molecule is present in the NCs. The previous study indicated that the OH groups of PVA was adsorbed on the surface of the NCs and can keep the NCs in a pseudo-hydrated state [36]. The diffraction patterns of the native PLA exhibit a broad diffraction peak from 2*θ *= 16.7° to 2*θ *= 35°. These results indicate that Doc is not present as a crystalline state, but is probably dissolved within the NCs' core and shell while some residual PVA might be present on the surface of the NCs.

#### In vitro drug release study

**Figure 6:**
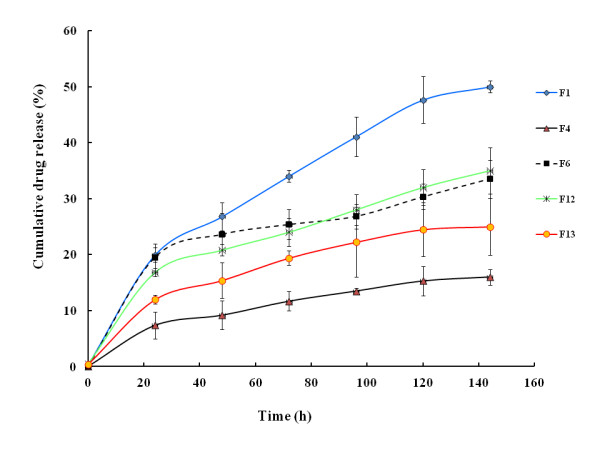
**Cumulative drug release from Doc-loaded nanocapsules in PBS (pH 7.4), at 37 ± 0.5°C**. Each point represents the mean value of three different experiments ± standard deviation.

The purpose of this study was to investigate the drug release mechanisms from Doc-loaded NCs. The cumulative percentages of Doc released from NCs as a function of time are reported in Figure 6. The results indicate that the drug release rate depends on the NCs' properties. The Doc oily core NCs were characterized by a sustained release profile (less than 40% of Doc is released within 60 h). This might be attributed to the hydrophobic interaction between the hydrophobic moiety of PLA, oil, and Doc. Compared to a previous result using nanosphere-based PLA [37], one can assume that the drug release is mainly due to the oil contained inside the NCs' core. The release data of Doc from the NCs (Figure 6) were fitted to the Higuchi model [38] to determine the drug release mechanism. The drug release constant (*k*) and regression coefficient (*R*^2^) of the Higuchi model are shown in Table S2 in Additional file 1. Accordingly, the Doc release was best supported by Higuchi's model, i.e., based on Fickian diffusion, as it presented the highest values of linearity (*R*^2 ^> 0.93) for all formulations. From F_6_, the release rate of Doc in 24 h corresponds to 0.9 nmol. This concentration is consistent with a previous report where the EC_50 _of Doc was ranged from 1 to 6.2 nmol. [39]. In these conditions, the low drug level in simulated plasma pH suggests that a triggering mechanism would be required to enhance drug release *in situ *within the targeted cancer cells in order to spare vital organs and significantly reduce the unwanted systemic side effect. To better understand the compatibility between Doc and PLA, as well as the neutral oil, the Flory-Huggins interaction parameter calculations were carried out as shown below.

#### Solubility parameter determination

To prove the suitability of PLA and oil for the optimal solubilization of Doc in the nanostructure, the Flory-Huggins interaction parameters were computed. The solubility parameters of Doc (*δ*_s_) and PLA (*δ*_p_) were calculated and listed in Table S3 in Additional file 1. According to the Flory-Huggins theory, the critical *χ*_sp _value above which a polymer and a low molecular weight compound (i.e., drug) become miscible is < 0.5. Therefore, a lower value of *χ*_sp _should result in a higher solubility. The obtained interaction parameters (*χ*_sp_) between Doc and PLA and between Doc and oil were calculated. Table S3 in Additional file 1 shows a low value of the interaction parameter (χ_sp_) between Doc and PLA 0.65 (cal/cm^3^)^1/2^. In contrast, the interaction solubility value between Doc/oil was relatively high 1.64 (cal/cm^3^)^1/2^, indicating that Doc is more compatible with PLA than with the oil. This finding seems to show that a high PLA content may contribute to high drug EE% (up to 93%). To confirm the suitability of the oil/ethyl acetate mixture (phase A) with water (phase B) for the NCs' formation, the interfacial tension (*γ*_AB_) between these two immiscible phases (A and B) was predicted by Kim and Burgess [40] using Equation 7:

(7.1) $$\gamma_{\text{AB}}=\gamma_{\text{A}}-\gamma_{\text{B}}\;\;\exp\;\left(-\alpha V^{0.7}\right)+\gamma_{\text{B}}$$

where *γ*_A_and *γ*_B _(72 mN/m at 25°C) are the surface tension of each phase (A or B), *α *is an exponential coefficient, and *V *is the volume fraction of the oil mixture. The *α*-value can be calculated by using the following equation:

(8) $$\alpha =0.178\;\left(\Delta \gamma \right)-0.0132$$

where Δ*γ *is the surface tension difference between ethyl acetate and oil, respectively.

The surface tensions of pure oil and ethyl acetate are 30.00 mN/m [41] and 6.80 mN/m [42], respectively. The Δ*γ *resulting from mixing these two substances is 23.20 mN/m, which is estimated to be *γ*_A_. From Equation 8, *α *was found to be 4.10 mN/m. However, the obtained value of *γ*_AB _from Equation 7 was 7.94 mN/m.

It is important to bear in mind that the interfacial tension of the oil/water system could be altered when another organic liquid is added to the oil phase, resulting in a compositional change at the interface and hence changes in the cohesive and adhesive forces [40]. On the basis of the above result, it is useful to rationalize whether or not the oil droplet entrapment/engulfing inside the polymer shell can occur. To be effective, this should normally occur before the solvent diffusion step, where the polymer forming the shell is in a liquid state. To achieve this goal, the oil droplet inclusion within the polymer shell has been predicted using the interfacial tension between the three phases, A, B, and C, and the spreading coefficients as defined [43,44]:

(9) $$S_{\text{A}}=\gamma_{\text{BC}}-\left(\gamma_{\text{AB}}+\gamma_{\text{AC}}\right)$$

(10) $$S_{\text{B}}=\gamma_{\text{CA}}-\left(\gamma_{\text{BC}}+\gamma_{\text{BA}}\right)$$

(11) $$S_{\text{c}}=\gamma_{\text{AB}}-\left(\gamma_{\text{CB}}+\gamma_{\text{CA}}\right)$$

where *γ *is the interfacial tension (A, B, and C refer to the three phases). Based on these conventions, complete engulfing of phase A by phase C will occur if only if *S*_A _< 0, *S*_B _< 0 and *S*_c _> 0. In this study, oil-ethyl acetate mixture is the phase A, water is phase B, and the polymeric phase (PLA) is phase C. The calculation of the spreading coefficients of a specific phase A/phase C system allows predicting the possible formation of oily core NCs.

The corresponding interfacial tension values were obtained as follows: *γ*_BC _was obtained from the literature (*γ*_BC _= +6.83 mN/m [45], *γ*_AC _= +0.06 mN/m was obtained from Table S4 in Additional file 1[46,47], and *γ*_AB _calculated from Equation 7 was 7.94 mN/m, which is comparable to the literature value of 8.42 mN/m [45]. The positive sign of the water-PLA interfacial tension (*γ*_BC _= +6.83 mN/m) implies that it tries to reduce its energy by reducing its surface area, and therefore, a spherical shape might be maintained. Based on these data, the obtained spreading coefficients were *S*_A _= -1.17 mN/m (< 0), *S*_B _= -14.71 mN/m (< 0), and *S*_C _= +1.05 mN/m (> 0). Thermodynamically, the driving forces allowed the formation of a PLA layer between the water and oily phase, thus engulfing the oily phase. These data fundamentally explain why the oily core was surrounded by a polymer layer as visually evidenced by the TEM analysis.

#### In vitro evaluation of Doc-loaded NC cytotoxicity

**Figure 7:**
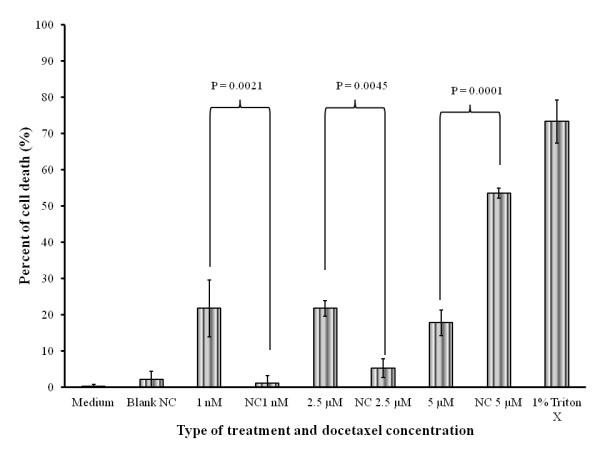
**Percent SUM 225 cell death by LDH assay after different treatments for 24 h**. See text for details. Pairwise comparison: native drug versus drug loaded NCs at the same concentration. Data are expressed as mean percent ± SD from six measurements (*n *= 6).

Figure 7 shows the cytotoxicity from the LDH assay of native Doc and Doc-loaded NCs at three tested different concentrations. The cell culture medium used as a negative control was not cytotoxic. At lower concentrations, the native drug appeared more bioactive perhaps due to rapid diffusion and higher level of interaction with the cells. However, at the highest concentration (5 μM), the drug-loaded NCs' cytotoxicity was significantly higher than that of the native Doc. This data clearly suggests that the oily core NC formulation (as a reservoir system for the drug) could enhance the biological responses of the native Doc with higher drug payload. This might be due to the sustained release properties of the NCs and the enhanced drug internalization by SUM 225 cells in the nanocapsule form. This observation was consistent with a previous report [48] related to a nanoparticulate form of another anticancer drug (doxorubicin) tested on a different breast cancer cell line (MCF7).

## Conclusions

In this work, we developed PLA-based Doc-loaded oily NCs by solvent diffusion method. The results confirmed the formation of Doc-loaded oily core polyester-shell NCs in the nanosize range (115 to 582 nm) and high EE% (65% to 93%). Most of the NCs were monodisperse in size (PDI = 0.005) with smooth surface. The PXRD data suggested that Doc was dissolved in the NCs. The Doc release data over 144 h fitted well with the Higuchi model (*R*^2 ^> 0.93), indicating that the drug mainly diffused out of the NCs in this timeframe. Consistent with the analysis of the spreading coefficients and visual evidence from EM, the NCs' oily core was indeed formed. The results from the cytotoxicity study suggested that at a high concentration (5 μM), the enhanced toxicity of the encapsulated drug on the examined cancerous cell line might be due to both the particle's uptake by the SUM 225 cells and the sustained drug release profile from the NCs. In a future work, we plan to investigate the cancer cell targeting capability of pegylated Doc-loaded NCs conjugated to specific ligands for drug delivery applications.

## Competing interests

The authors declare that they have no competing interests.

## Authors' contributions

IY designed the present work and carried out most of the experimental work (including the preparation and characterizations of the sample and the analysis of experimental data). XY participated in the analysis of the particle size, morphology, and PXRD spectra. JBM carried out the PXRD analysis. BBCY participated in the design of the study and coordination of the work as lead investigator. All authors contributed to the interpretation of the results and the drafting of the manuscript, and they read and approved the final version.

## Authors' information

IY has a Ph.D. degree in Pharmaceutical Sciences and is a post doctoral research associate in the Division of Pharmaceutical Sciences, School of Pharmacy, University of Missouri-Kansas City. XY is a graduate student in the Division of Pharmaceutical Sciences, School of Pharmacy, University of Missouri-Kansas City. JM has BS, MS, and Ph.D. degrees in Geochemistry and Mineralogy. He is an associate professor in the Department of Geosciences, University of Missouri-Kansas City. BBCY has PharmD and Ph.D. degrees in Pharmaceutical Sciences. He is also an associate professor in the Division of Pharmaceutical Sciences, School of Pharmacy, University of Missouri-Kansas City.

## Supplementary Material

Additional file 1 **Supplementary tables**. Four supplementary tables showing the physicochemical characteristics of the Doc-loaded oily core nanocapsules (*n *= 3), key parameters resulting from fitting the Doc release profile to the Higuchi model, physicochemical properties of drug and excipients, and physicochemical properties and surface tension of drug and excipients, respectively. Click here for file
