# Supplementary material for: Encapsulation of docetaxel in oily core polyester nanocapsules intended for breast cancer therapy
Source: Nanoscale Res Lett. 2011 Dec 14;6(1):630. doi: 10.1186/1556-276X-6-630 (PMC3292599; doi:10.1186/1556-276X-6-630)
Supplement: Additional file 1 — Supplementary tables. Four supplementary tables showing the physicochemical characteristics of the Doc-loaded oily core nanocapsules (n = 3), key parameters resulting from fitting the Doc release profile to the Higuchi model, physicochemical properties of drug and excipients, and physicochemical properties and surface tension of drug and excipients, respectively. [file 1556-276X-6-630-S1.DOC]

**Table 1**. Physicochemical characteristics of the Doc-loaded oily core nanocapsules (n=3).

| Run No | PLA | Doc | oil | Actual Doc loading (%, w/w) | Mean diameter (nm) ± S.D.^*^ | Polydispersity index | EE%± S.D.^*^ |
| --- | --- | --- | --- | --- | --- | --- | --- |
| 1 | 40 | 10 | 0.9 | 3.3 | 402.6±21.2 | 0.296 | 74.4±1.0 |
| 2 | 200 | 10 | 0.5 | 26.9 | 141.8±2.0 | 0.005 | 91.3±1.1 |
| 3 | 360 | 10 | 0.1 | 68.3 | 189.5±2.7 | 0.005 | 90.2±2.4 |
| 4 | 360 | 18 | 0.5 | 39.2 | 134.7±1.8 | 0.005 | 92.7±1.5 |
| 5 | 360 | 2 | 0.5 | 39.6 | 179.1±2.0 | 0.005 | 91.8±1.7 |
| 6 | 200 | 18 | 0.9 | 17.5 | 115.6±1.3 | 0.005 | 93.5±2.5 |
| 7 | 200 | 2 | 0.9 | 17.7 | 119.0±1.4 | 0.005 | 93.2±2.7 |
| 8 | 200 | 10 | 0.5 | 26.4 | 196.7±2.8 | 0.005 | 90.0±1.4 |
| 9 | 40 | 10 | 0.1 | 16.8 | 582.8±28.6 | 0.318 | 65.3±3.1 |
| 10 | 40 | 2 | 0.5 | 6.7 | 239.2±10.4 | 0.261 | 85.9±1.9 |
| 11 | 40 | 18 | 0.5 | 6.3 | 272.4±9.7 | 0.261 | 84.0±2.8 |
| 12 | 200 | 10 | 0.5 | 27.0 | 154.2±2.4 | 0.005 | 92.0±2.4 |
| 13 | 360 | 10 | 0.9 | 27.4 | 133.3±1.9 | 0.005 | 92.9±3.0 |
| 14 | 200 | 18 | 0.1 | 51.6 | 247.1±2.5 | 0.004 | 83.6±1.7 |
| 15 | 200 | 2 | 0.1 | 47.8 | 438.3±23.7 | 0.269 | 73.5±2.2 |

**Table 2:** Key parameters resulting from fitting Doc release profile to Higuchi model

|  | **Higuchi equation** | |
| --- | --- | --- |
|  | R² | K**_H_** |
| F1 | 0.998 | 5.787 |
| F2 | 0.953 | 1.836 |
| F3 | 0.955 | 1.781 |
| F4 | 0.996 | 0.272 |
| F5 | 0.933 | 2.685 |
| F12 | 0.993 | 2.46 |
| F13 | 0.992 | 1.843 |
| F14 | 0.964 | 2.180 |

**Table 3.** Physico-chemical properties of drug and excipients.

| Chemicals | Mw (Da) | Vm (cm^3^/mol) | δ (Mpa)^1/2^ | Interaction parameter with DOC [χ_sp_, cal/cm^3^)^½^] |
| --- | --- | --- | --- | --- |
| Doc | 807.90ª | 664.40^b^ | 27.14^c^ | - |
| PLA | 250000 | 79.98^b^ | 18.20 ^c^ | 0.65 |
| ETAc | 88.10 | 98.50^b^ | 18.15 ^c^ | - |
| Labrafac CC (Oil) | NA | NA | NA | 1.64 |
|  |  |  |  |  |

1. Note: Huynh et al. 2008, b) Molar volume (Vm) of excipients was calculated from their density and the molecular weight. c) solubility parameter were obtained from Hansen, 1967.

**Table 4.** Physico-chemical properties and surface tension of drug and excipients

|  | MW | density (g/cm^3^) | Surface tension (Nm/m) | Mole fraction (mole) |
| --- | --- | --- | --- | --- |
| oil | 316.47 | 0.945 | 30^a^ | 8.49x10^-6^ |
| Ethyl acetate | 88 | 0.897 | 6.8^b^ | 1.16x10^-3^ |
| PLA (C) | 72 | - | 36^c^ | 1.34x10^-3^ |
| Water (B) | 18 | 1 | 71.97^d^ | 2.5x10^-3^ |

A

^a^ Adapted from reference (Adamson and Gast, 1997)

^b^ Adapted from reference (Kobayashi et al, 2005)

c Adapted from reference (Biresaw and Carriere, 2002).

^d^ Adapted from reference (Kirchner, 2010).

Note: The density value and molecular for each liquid: ethyl acetate, water and Labrafac cc oil is based on manufacturer’s data sheet.

.
